# The network characteristics in schizophrenia with prominent negative symptoms: a multimodal fusion study

**DOI:** 10.1038/s41537-023-00408-2

**Published:** 2024-01-17

**Authors:** Li Kong, Yao Zhang, Xu-ming Wu, Xiao-xiao Wang, Hai-su Wu, Shuai-biao Li, Min-yi Chu, Yi Wang, Simon S. Y. Lui, Qin-yu Lv, Zheng-hui Yi, Raymond C. K. Chan

**Affiliations:** 1 Department of Psychology, Shanghai Normal University, Shanghai, China; 2 Shanghai Mental Health Centre, Shanghai Jiao Tong University School of Medicine, Shanghai, China; 3 Department of Psychiatry, Huashan Hospital, Fudan University, Shanghai, China; 4 Nantong Fourth People’s Hospital, Nantong, China; 5 Neuropsychology and Applied Cognitive Neuroscience Laboratory, CAS Key Laboratory of Mental Health, Institute of Psychology, Chinese Academy of Sciences, Beijing, China; 6 Department of Psychology, University of Chinese Academy of Sciences, Beijing, China; 7 Department of Psychiatry, School of Clinical Medicine, The University of Hong Kong, Hong Kong Special Administrative Region, China; 8 Institute of Mental Health, Fudan University, Shanghai, China

**Keywords:** Psychiatric disorders, Diseases

## Abstract

Previous studies on putative neural mechanisms of negative symptoms in schizophrenia mainly used single modal imaging data, and seldom utilized schizophrenia patients with prominent negative symptoms (PNS).This study adopted the multimodal fusion method and recruited a homogeneous sample with PNS. We aimed to identify negative symptoms-related structural and functional neural correlates of schizophrenia. Structural magnetic resonance imaging (sMRI) and resting-state functional MRI (rs-fMRI) were performed in 31 schizophrenia patients with PNS and 33 demographically matched healthy controls.Compared to healthy controls, schizophrenia patients with PNS exhibited significantly altered functional activations in the default mode network (DMN) and had structural gray matter volume (GMV) alterations in the cerebello-thalamo-cortical network. Correlational analyses showed that negative symptoms severity was significantly correlated with the cerebello-thalamo-cortical structural network, but not with the DMN network in schizophrenia patients with PNS.Our findings highlight the important role of the cerebello-thalamo-cortical structural network underpinning the neuropathology of negative symptoms in schizophrenia. Future research should recruit a large sample and schizophrenia patients without PNS, and apply adjustments for multiple comparison, to verify our preliminary findings.

## Introduction

Schizophrenia is a heterogeneous disorder with varied symptoms, and its neurobiological mechanisms remain unclear^1^. Negative symptoms are refractory to treatments and often associated with poor functional outcomes^2,3^. According to the Research Domain Criteria (RDoC), clarifying the underlying mechanisms for negative symptoms is important to unveil the etiological mechanisms for schizophrenia^4^.

Previous studies have provided scattered evidence on structural and functional abnormalities responsible for negative symptoms of schizophrenia. Structural magnetic resonance imaging (sMRI) studies showed that severity of negative symptoms was correlated with gray matter volume (GMV) reduction in the prefrontal^5,6^ and temporal cortices^7^, and the paracingulate^8^. Findings from positron emission tomography (PET) and functional magnetic resonance imaging (fMRI) showed neural functional correlation of negative symptoms in schizophrenia. For instance, PET studies showed that negative symptoms were associated with decreased activations in the medial frontal gyrus but increased activations in the left temporal cortex in schizophrenia patients^9–11^. Moreover, fMRI studies reported associations of negative symptoms with altered functional connectivity (in particular the default mode network, DMN), and activation in the prefrontal and temporal cortices as well as the ventral striatum^12–14^. However, inconsistent findings have been found for putative brain structural and functional correlates of negative symptoms. For instance, some studies failed to find any correlation of GMV or brain functional alterations with negative symptoms in schizophrenia patients^15,16^, while other studies reported increased GMV in the prefrontal cortex and its association with negative symptoms severity in schizophrenia patients^17^. The divergent findings in the extant literature may be attributable to (1) heterogeneity in samples and (2) variations in imaging analysis methods. To address phenotypic heterogeneity, recent studies had recruited homogeneous sample, such as schizophrenia patients with predominant or persistent negative symptoms^18–21^, and found that such schizophrenia subgroups showed significantly abnormal GMV or functional activation in the prefrontal and temporal cortex, the insula, basal ganglia, thalamus and cerebellum.

Although several studies utilized homogeneous schizophrenia sample, they only adopted single modal analysis, which was unable to capture the full spectrum of brain abnormalities. Recent multimodal imaging studies suggested that multimodal analysis is a promising approach to elucidate shared disease factors across different modalities for constructing plausible “biologically convergent” models^22–27^. Such “multimodal fusion approach” could be applied to unveil the neurobiological processes for schizophrenia^28^, and could take into account the interrelationship between voxels to identify network changes that single modal analyses could not detect.

In this study, we adopted a multiset canonical correlation analysis together with the joint independent component analysis (mCCA + jICA) to identify structural and functional networks across multimodalities. Moreover, this study utilized a homogeneous schizophrenia sample with prominent negative symptoms (PNS). The mCCA + jICA, a multimodal fusion approach, can simultaneously decompose multiple modalities of data and maximize the inter-modality covariance to identify maximally independent components across modalities as well as subject-specific weights on the group-level independent components^24,25,29^. Two represented MRI features from multiple modalities, i.e., (1) the fractional amplitude of low-frequency fluctuations (fALFF) gathered using rs-fMRI, and (2) the GMV gathered using sMRI, were combined to generate a fusion model, which yield modality-linked independent components at group-level and individual subject weightings on these components. Therefore, this approach allowed us to investigate the relationships between structural and functional brain networks, associated with negative symptoms. We hypothesized that schizophrenia patients with PNS would exhibit structural and functional network abnormalities, which partly differ from those found in schizophrenia patients using single modal approach. In addition, we hypothesized that the identified structural and functional networks would be associated with negative symptoms in our sample with PNS schizophrenia.

## Materials and method

### Participants

Thirty-two schizophrenia patients with PNS were recruited from the Shanghai Mental Health Centre. Our clinical participants were deemed to have PNS if they were rated >3 on at least 3 items, or >4 on at least 2 items of the negative symptoms subscale of the Positive and Negative Syndrome Scale (PANSS; Kay, 1987), and were rated <19 on the PANSS positive symptoms subscale^30–32^. We excluded one schizophrenia patient due to excessive head motion (>2 mm translation) during brain scanning. Healthy controls were recruited from the neighboring community, using the exclusion criteria as follows: (1) personal history of psychiatric disorder, (2) neurological disease, (3) head injury resulting from loss of consciousness for >30 min, and (4) substance abuse in the past 6 months. The final sample comprised 31 schizophrenia patients with PNS (18 males; mean age = 24.23, SD = 4.73; mean education = 12.37, SD = 2.88) and 33 healthy controls (19 males; mean age = 25.85, SD = 2.53; mean education = 15.61, SD = 3.77). All participants were right-handed as assessed using the Edinburgh Handness Inventory^33^. Diagnosis was ascertained using the Structured Clinical Interview for DSM-5^34^. Clinical participants received second-generation antipsychotics (mean daily dose of chlorpromazine equivalence = 308.64 mg, SD = 174.13 mg), but 6 clinical participants’ medication data was not complete at the time of neuroimaging assessments. Psychopathological symptoms were measured using the PANSS^35^. This study was approved by the Ethics Committee of the Shanghai Mental Health Centre (Protocol no.: 2021-50). Written informed consent were obtained from all participants.

### MRI acquisition

Participants undertook structural brain scanning within a 3-Tesla MR scanner (Verio, Erlangen, Germany). High-resolution T1-weighted structural imaging data were acquired for anatomical reference through a magnetization-prepared rapid gradient-echo (MPRAGE) sequence (repetition time (TR) = 2530 ms, echo time (TE) = 1.66 ms, flip angle = 7°, field of view = 256 × 256 mm, image matrix = 256 × 256, voxel size = 1 × 1 × 1 mm^3^). For functional MRI, Planar echo imaging oxygen saturation dependence (ep2D bold) sequence scanning method was used. The specific scanning parameters were as follows: repeat time (TR) = 2600 ms; Echo time (TE) = 30 ms; Acquisition Matrix = 64 × 64; Flip Angle = 90°; Scan thickness (ST) = 3.5 mm; FOV = 448 × 448 mm; voxel size = 3.1 × 3.1 × 3.5 mm, 40 slices.

### Multimodal imaging preprocessing

Voxel-based morphometry of the structural brain images were preprocessed using an automated toolbox cat12 (https://neuro-jena.github.io/cat/) in SPM12 (https://www.fil.ion.ucl.ac.uk/spm/software/spm12/) that we described in our previous study^36^. In brief, the process first normalized the structural images to a Montreal Neurological Institute (MNI) template, and then segmented these normalized images into cerebrospinal fluid, white matter and gray matter^37^. We finally smoothed the segmented images of gray matter with 6-mm full width at half-maximum Gaussian kernel.

The functional brain images were preprocessed using the Data Processing Assistant for rs-fMRI (DPARSF)^38^. Considering the instability of the initial MRI signal and adaptation of participants to the circumstance, the first 10 volumes of each participant were discarded^39,40^. The remaining images were then processed with slice timing and head motion correction. Subsequently, the corrected images were then normalized to the standard MNI space. After that, white matter signals, cerebrospinal fluid and the Friston 24-parameters were regressed^41^. Finally, the images were smoothed with a 6-mm FWHM isotropic Gaussian kernel. After the preprocessing procedure, we estimated the ratio of total amplitude within the low-frequency range (0.01–0.08 Hz) to the entire detectable frequency range, as the fALFF^42^.

After extracting the GMV and fALFF imaging features, a multimodal “MCCA + jICA” model in the Fusion ICA Toolbox (http://mialab.mrn.org/software/fit) was performed for a fusion analysis^24,25,43^. All participants of each modality were reshaped into a feature matrix with rows representing subjects and columns representing voxels. The feature matrices within modality were normalized to have the same average sum of squares to ensure both modalities had the same range of values in each group that contribute equally in fusion analysis. After joint decomposition, independent components (ICs) and subject loadings for each modality were derived. For each modality, the number of components was estimated based on information theoretic criteria^44^. Twelve ICs were estimated for each modality in this study. To visualize the components, the source matrix was reshaped backed to a 3D-image and subsequently scaled to unit standard deviations (*z*). Thereafter, the image was thresholded at |z| > 2.5. Clusters of significance within ICs were identified by a spatial extent threshold of greater than 1 cubic centimeter in at least one hemisphere.

### Statistical analysis

We adopted the SPSS software version 25.0 to analyze clinical and demographic information between schizophrenia patients and healthy controls. We also performed analyses of covariance were to compare the ICs loading coefficients between schizophrenia patients and healthy controls. A significance level of *p* < 0.05 was chosen as the nominal significance threshold (uncorrected for multiple comparisons). Further correlation analyses were performed to examine the associations between the identified ICs and PANSS scores, with sex, gender and duration of illness as covariates.

## Results

### Demographic and clinical characteristic

Table 1 summarizes the characteristics of our sample. The clinical and the control groups did not differ in age (*p* = 0.097) and sex (*p* = 0.968), but differed in length of education (*p* < 0.001).

**Table 1:** Clinical and demographic information for the participants.

	Schizophrenia (*n* = 31)	Controls (*n* = 33)	*t*-value (df = 62)	*p* value
Age (years)	24.23 (4.73)	25.85 (2.53)	−1.70	0.097
Length of education (years)	12.37 (2.88)	15.61 (3.77)	−3.84	<0.001
Sex (male/female)	18/13	19/14		0.968^a^
Duration of illness (months)	57.58 (42.02)	–		–
Medication^b^ (CPZ equivalence, mg/day)	308.64 (174.13)	–		–
PANSS total score	77.32 (12.51)	–		–
PANSS positive score	12.58 (3.12)	–		–
PANSS negative score	26.94 (6.73)	–		–
PANSS global score	37.81 (6.50)	–		–

Data are mean ± SD.

*SD* standard deviation, *CPZ* chlorpromazine, *PANSS* Positive and Negative Syndrome Scale, *df* degrees of freedom.

^a^*χ*^2^-test

^b^There were six patients with missing data on medication doses and four patients were medication naïve.

### Different network characteristics

**Fig. 1:**
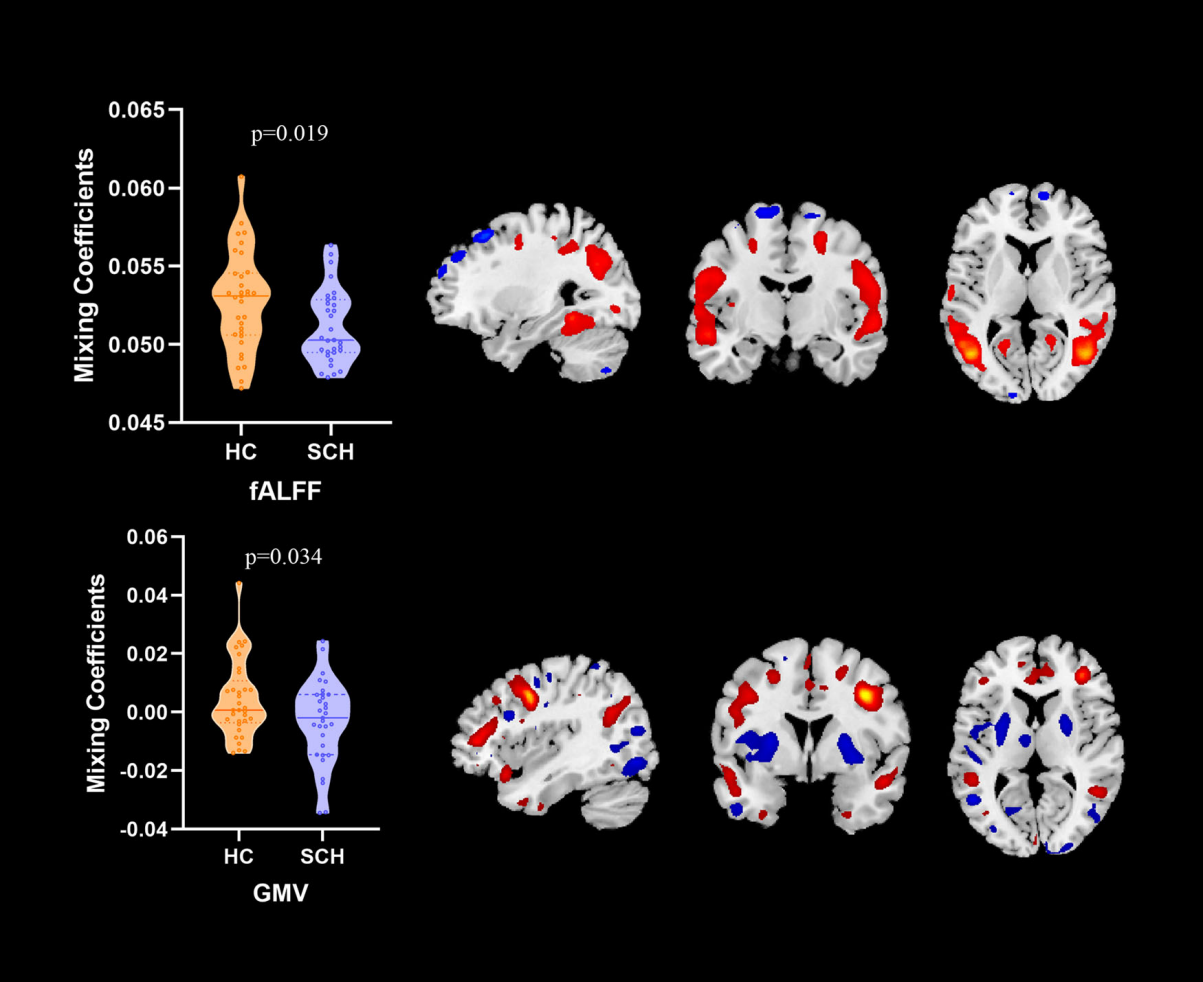
Group-discriminating regions across two modalities, with |z| > 2.5. Boxplot of the loading coefficients of both groups on the mean of loadings parameters for each modality. HC healthy controls, SCH schizophrenia, fALFF fractional amplitude of low-frequency fluctuations, GMV gray matter volume.

Figure 1 illustrates that the fusion model identified 2 modal-specific ICs showing significant differences in loading coefficients between schizophrenia participants with PNS and healthy controls. One IC from fMRI (DMN network) showed significantly reduced loading coefficients in schizophrenia participants with PNS, suggesting reduced activation, which mainly involved the bilateral superior/middle/inferior temporal cortices, the precuneus, the inferior parietal cortex, the posterior cingulate and prefrontal cortex, and increased loading coefficients in the bilateral superior/middle/medial frontal cortices (see Supplementary Table S1 for details). The other IC from sMRI (cerebello-thalamo-cortical network) showed significantly smaller loading coefficients in patients, suggesting reduced GMV, mainly involving in the bilateral superior/middle temporal cortices, the medial/middle/inferior prefrontal cortices, the anterior cingulate, and larger loading coefficients in schizophrenia participants with PNS, which mainly involved the bilateral superior/middle/inferior frontal cortices, the thalamus and the cerebellum (see Supplementary Table S1 for details).

### The associations between identified networks and PANSS scores

**Fig. 2:**
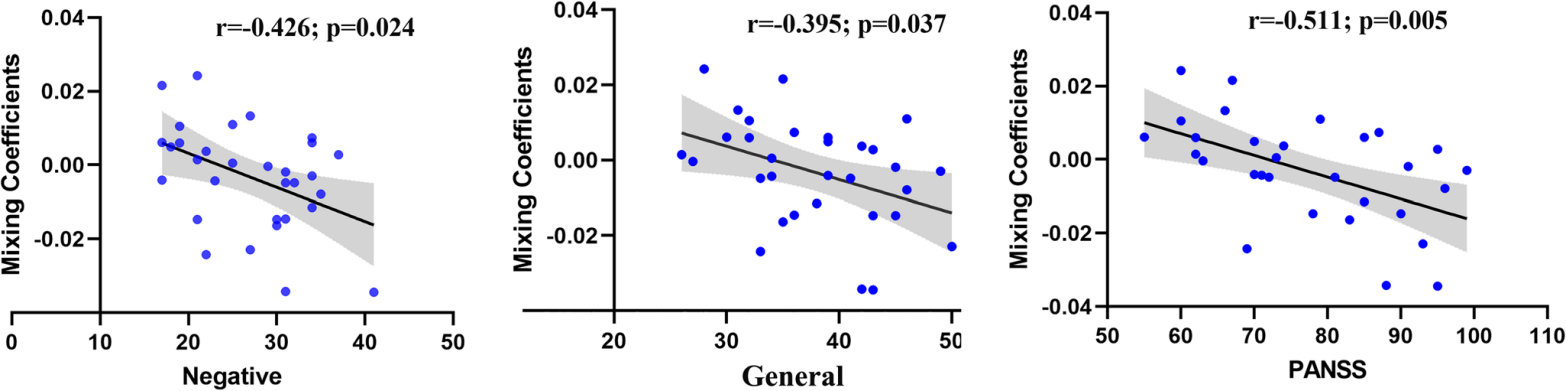
The scatter plots and linear trends of components with significant correlation between PANSS score and its loadings. PANSS Positive and Negative syndrome Scale.

Correlational analyses examined the associations between the identified networks and the PANSS scores, with age, gender and duration of illness as covariates. We found that the cerebello-thalamo-cortical network abnormalities from sMRI were associated with PANSS negative symptoms (*r* = −0.426, *p* = 0.024), PANSS general psychopathology symptoms (*r* = −0.395, *p* = 0.037) and the PANSS total score (*r* = −0.511, *p* = 0.005) (see Fig. 2). However, we did not find any significant correlation between the fMRI DMN network and the PANSS total as well as subscale scores. When we further tested the correlations of the fMRI DMN network with each of the PANSS negative symptom items, we found that the DMN network was associated with PANSS item of blunted affect (*r* = 0.398, *p* = 0.036). However, after adjustments for multiple comparisons, all these significant correlation results disappeared.

To account for the possible confound of the group difference in education, we examined the group differences in networks as well as their associations with PANSS, by including age, gender, duration of illness and education as covariates. Our covariate results remained similar and significant, except one result of group difference in structural network become weaker (see Supplementary Materials for details).

## Discussion

This study investigated the neural correlates of negative symptoms in schizophrenia patients with PNS using the multimodal neuroimaging fusion analysis approach, which incorporated sMRI and rs-fMRI data. Our study has three key findings. First, the rs-fMRI identified regions within the DMN network where altered neural activity could be found in schizophrenia patients with PNS relative to healthy controls. Second, the sMRI identified regions of the cerebello-thalamo-cortical network where altered GMV could be found in schizophrenia patients with PNS relative to healthy controls. Third, the identified structural cerebello-thalamo-cortical network abnormalities were associated with negative symptoms of schizophrenia.

The findings of alterations within DMN network in schizophrenia patients with PNS are consistent with previous fMRI findings showing altered DMN activation or connectivity in schizophrenia^45–47^, supporting these neural correlates as a putative neurobiological marker for schizophrenia. Moreover, our findings of decreased neural activation in the posterior DMN concur with a recent multimodal study reporting a lower fALFF in the posterior DMN in schizophrenia^48^. However, differing from Sui et al.^48^, we found increased neural activation in the prefrontal cortex. Although schizophrenia has been consistently associated with abnormal functions of the prefrontal cortex, it remains unclear whether the prefrontal cortex would exhibit increased or decreased neural activities, because previous findings were inconsistent^45,46,49–51^. The divergent findings might be related to sample heterogeneity and methodological issues (such as the use of regions of interest (ROI) or independent component analysis (ICA)). Our work benefited from the use of multimodal fusion method and a homogeneous sample of schizophrenia with PNS.

Some studies reported altered functional activations in the DMN were associated with negative symptoms in schizophrenia^14,46^. A recent study investigating dynamic functional network changes in schizophrenia patients showed variability of the dynamic segregating process in the DMN, which was negatively related to avolition symptom in schizophrenia^14^. However, the identified functional component of the DMN in our sample was not significantly associated with the PANSS negative symptoms, albeit its association with the PANSS item of blunted affect was significant. Given that the PANSS is a conventional rating for negative symptoms, this scale may be insufficient to assess the complex psychopathology of negative symptoms^35^. Future research should clarify the association of the DMN network with negative symptom using second-generation negative symptom scales such as the Brief Negative symptom Scale^52^ and the Clinical Assessment Interview for Negative Symptoms^53^ in a large sample of schizophrenia patients.

Regarding the structural components, we identified that schizophrenia patients with PNS showed abnormalities in the cerebello-thalamo-cortical network. In fact, altered cerebello-thalamo-cortical circuits in schizophrenia patients has generally been well-supported in the literature, and such neural correlates appeared to be independent of the illness stage^54–60^ and medication confounds^61^. In addition, a recent study proposed a multimodal classification to classify schizophrenia patients and healthy controls by combined structural and functional features, and found that the most distinguishing features between the two groups included the cerebello-thalamo-cortical circuits^62^. Taken together, pathological processes in the cerebello-thalamo-cortical connectivity may be a putative biomarker for schizophrenia.

Our findings also suggested that the abnormal cerebello-thalamo-cortical networks were associated with negativity symptoms severity in schizophrenia patients with PNS. Partly consistent with our results, a recent fMRI study reported that cerebello-thalamo-cortical connectivity disturbances in schizophrenia patients were not directly correlated with negative symptoms at baseline but correlated with the changes of negative symptoms in the long term^61^. Longitudinal study could reduce the confounding effect of intra-individual heterogeneity by using each subject as his or her own control. Cao et al.’s^61^ findings indicated that the cerebello-thalamo-cortical connectivity disturbances may predict negative symptoms-associated functional outcomes in schizophrenia patients. Our preliminary findings suggested that abnormal structural networks in cerebello-thalamo-cortical circuits may constitute an “anatomical basis” for the functional abnormality in cerebello-thalamo-cortical connectivity in schizophrenia patients. Interestingly, another study using repetitive transcranial magnetic stimulation (TMS) had targeted at the cerebellar-prefrontal connectivity in schizophrenia patients and reported reduced negative symptoms after TMS treatment^63^. Taken together, dysfunctional connectivity of cerebello-thalamo-cortical network may play a critical role in the pathophysiology of the negative symptoms in schizophrenia.

This study has several notable limitations. First, our sample size was very small, and we did not adjust for multiple comparisons, otherwise our findings would become non-significant. The use of such a small sample did not allow use to apply stringent statistical analyses for adjusting multiple comparisons. However, our unadjusted findings converged, and were generally consistent with the previous literature showing altered DMN network^46^ and cerebello-thalamo-cortical network disturbances^58^ in schizophrenia. Third, we did not recruit schizophrenia patients without PNS as a comparison group. Future study should include well-matched schizophrenia groups with and without PNS to examine whether the identified imaging features in our study would be specific to schizophrenia patients with PNS. Fourth, some of our clinical participants received antipsychotic medication. The potential confounds of antipsychotic medication on brain networks should be further clarified in future study.

To conclude, our preliminary findings suggested that schizophrenia patients with PNS showed aberrant DMN functional network and disrupted cerebello-thalamo-cortical structural network. Altered cerebello-thalamo-cortical network may be a putative biomarker for negative symptoms in schizophrenia.

### Supplementary information

supplementary materials

## Data Availability

The data of this study are available from the corresponding author upon reasonable request.
