# Author Correction: The network characteristics in schizophrenia with prominent negative symptoms: a multimodal fusion study

**DOI:** 10.1038/s41537-024-00467-z

**Published:** 2024-04-08

**Authors:** Li Kong, Yao Zhang, Xu-ming Wu, Xiao-xiao Wang, Hai-su Wu, Shuai-biao Li, Min-yi Chu, Yi Wang, Simon S. Y. Lui, Qin-yu Lv, Zheng-hui Yi, Raymond C. K. Chan

**Affiliations:** 1 Department of Psychology, Shanghai Normal University, Shanghai, China; 2 Shanghai Mental Health Centre, Shanghai Jiao Tong University School of Medicine, Shanghai, China; 3 Department of Psychiatry, Huashan Hospital, Fudan University, Shanghai, China; 4 Nantong Fourth People’s Hospital, Nantong, China; 5 Neuropsychology and Applied Cognitive Neuroscience Laboratory, CAS Key Laboratory of Mental Health, Institute of Psychology, Chinese Academy of Sciences, Beijing, China; 6 Department of Psychology, University of Chinese Academy of Sciences, Beijing, China; 7 Department of Psychiatry, School of Clinical Medicine, The University of Hong Kong, Hong Kong Special Administrative Region, China; 8 Institute of Mental Health, Fudan University, Shanghai, China

**Keywords:** Psychiatric disorders, Diseases

Correction to: *Schizophrenia* 10.1038/s41537-023-00408-2, published online 17 January 2024

The Acknowledgements section was missing from this article and should have read “This study was supported by the Natural Science Foundation of China (82071501) and the Shanghai Science and Technology Innovation Action Plan Natural Science Fund Project (21ZR1455400). The original article has been corrected.”.

