# Supplementary material for: The network characteristics in schizophrenia with prominent negative symptoms: a multimodal fusion study
Source: Schizophrenia (Heidelb). 2024 Jan 17;10(1):10. doi: 10.1038/s41537-023-00408-2 (PMC10851703; doi:10.1038/s41537-023-00408-2)
Supplement: Supplementary file 1 — supplementary materials [file 41537_2023_408_MOESM1_ESM.doc]

**Supplementary Materials**

Table S1. Anatomical information of the identified components for schizophrenia and healthy controls

| **fMRI_fALFF Area** | **Brodmann Area** | **volume(cc)** | **random effects: Max (x,y,z)L/R** |
| --- | --- | --- | --- |
| **HC>SCH** |  |  |  |
| Middle Temporal Gyrus | 19, 21, 22, 37, 39 | 11.5/11.5 | 5.6 (-45, -64, 6)/5.6 (48, -64, 6) |
| Superior Temporal Gyrus | 13, 21, 22, 39, 42 | 7.0/6.9 | 4.8 (-50, -55, 11)/4.2 (48, -40, 10) |
| Inferior Temporal Gyrus | 19, 21, 37 | 1.0/0.8 | 4.6 (-50, -61, -2)/4.5 (45, -67, 1) |
| Precuneus | 7, 18, 19, 31, 39 | 5.8/6.3 | 4.2 (-6, -74, 26)/4.4 (21, -71, 39) |
| Postcentral Gyrus | 2, 3, 40, 43 | 4.5/3.5 | 3.8 (-59, -13, 23)/4.2 (42, -27, 37) |
| Middle Occipital Gyrus | 19, 37 | 4.5/2.9 | 5.1 (-42, -64, 3)/4.7 (48, -67, 3) |
| Inferior Parietal Cortex | 7, 39, 40 | 3.1/1.3 | 3.9 (-39, -30, 37)/4.0 (36, -47, 44) |
| Posterior Cingulate | 30, 31 | 1.5/1.5 | 3.8 (-21, -58, 6)/3.6 (21, -58, 6) |
| Cuneus | 7, 18, 19 | 3.5/1.9 | 4.7 (-6, -78, 23)/3.5 (27, -74, 31) |
| Parahippocampal Gyrus | 19, 30, 36, 37 | 1.1/1.7 | 4.0 (-24, -47, -8)/4.4 (27, -47, -10) |
| Lingual Gyrus | 18, 19 | 1.7/2.1 | 4.2 (-18, -52, 0)/4.0 (18, -49, 2) |
| Cerebellum | / | 3.3/3.2 | 4.2 (-18, -56, -7)/3.9 (21, -47, -10) |
| Fusiform Gyrus | 19, 20, 37 | 1.7/3.3 | 3.8 (-24, -50, -10)/4.0 (30, -47, -8) |
| Middle Frontal Gyrus | 6 | 0.9/1.0 | 4.0 (-24, -1, 44)/3.6 (27, -1, 47) |
| Precentral Gyrus | 3, 4, 6, 43 | 2.4/3.4 | 3.7 (-56, -13, 26)/3.8 (56, -13, 26) |
| Declive | / | 0.8/1.4 | 3.6 (-18, -59, -10)/3.6 (24, -53, -12) |
| **SCH>HC** |  |  |  |
| Superior Frontal Gyrus | 6, 8, 9, 10 | 12.9/13.2 | 6.2 (-21, 56, 22)/4.4 (18, 46, 36) |
| Middle Frontal Gyrus | 6, 8, 9, 10 | 7.4/3.8 | 5.6 (-24, 56, 19)/4.3(33, 26, 46) |
| Medial Frontal Gyrus | 6, 8, 9, 10 | 1.1/1.5 | 3.7 (-9, 48, 36)/3.6 (9, 48, 36) |
| Precentral Gyrus | 6, 9 | 1.0/0.0 | 3.9 (-48, -1, 47)/-999.0 (0, 0, 0) |
| **sMRI_GMV Area** | **Brodmann Area** | **volume(cc)** | **random effects: Max (x,y,z)L/R** |
| **HC>SCH** |  |  |  |
| Middle Temporal Gyrus | 20, 21, 22, 37, 38, 39 | 7.5/7.4 | 6.6 (-50, -25, -4)/7.1 (50, -52, 7) |
| Superior Temporal Gyrus | 21, 22, 38, 39 | 4.8/4.9 | 6.3 (-31, 13, -25)/7.1 (40, 19, -19) |
| Middle Frontal Gyrus | 6, 8, 9, 10, 11, 46 | 4.2/6.2 | 6.9 (-36, 32, 27)/11.2 (36, 6, 40) |
| Inferior Frontal Gyrus | 6, 9, 44, 46, 47 | 3.3/4.5 | 6.9 (-49, 8, 16)/9.1 (43, 4, 32) |
| Medial Frontal Gyrus | 6, 8, 9, 10, 11, 25 | 2.8/2.0 | 4.8 (-10, 46, -10)/3.6 (9, 47, 5) |
| Anterior Cingulate | 10, 24, 32 | 2.9/2.0 | 5.6 (-12, 47, -2)/4.7 (12, 44, 5) |
| Cingulate Gyrus | 24, 31, 32 | 1.2/2.8 | 4.2 (0, 4, 43)/4.8 (13, -27, 40) |
| Precuneus | 7, 19, 31, 39 | 1.3/1.1 | 4.2 (-27, -54, 52)/5.2 (40, -65, 35) |
| Precentral Gyrus | 6, 9 | 1.3/1.0 | 6.1 (-39, 3, 36)/13.2 (39, 6, 37) |
| Fusiform Gyrus | 20, 37 | 1.8/0.4 | 6.9 (-46, -41, -12)/4.0 (36, -40, -16) |
| Parahippocampal Gyrus | 34, 36, 37 | 0.6/1.5 | 4.0 (-22, 6, -18)/4.6 (31, -10, -26) |
| Parahippocampal Gyrus | 34, 36, 37 | 0.6/1.5 | 4.0 (-22, 6, -18)/4.6 (31, -10, -26) |
| **SCH>HC** |  |  |  |
| Superior Frontal Gyrus | 6, 8, 9, 10 | 4.7/2.7 | 6.1 (-13, 36, 47)/4.7 (16, 48, 29) |
| middle Frontal Gyrus | 6, 8, 10, 46 | 1.3/2.2 | 5.7 (-42, 20, 24)/5.5 (36, 44, 13) |
| inferior Frontal Gyrus | 9, 13, 45, 46, 47 | 1.2/0.6 | 5.9 (-49, 18, 10)/ 3.6 (46, 33, -10) |
| Cerebellum | * | 1.5/1.3 | 4.6 (-10, -48, 1)/4.1 (3, -50, -3) |
| Precentral Gyrus | 6, 13, 44 | 1.0/2.0 | 3.8 (-45, 0, 8)/4.8 (46, 1, 46) |
| Inferior Parietal Cortex | 7, 39, 40 | 1.6/0.8 | 6.3 (-33, -59, 42)/6.9 (36, -55, 39) |
| Thalamus | * | 1.6/0.6 | 4.0 (-9, -10, 7)/3.2 (12, -11, 12) |
| Insula | 13 | 1.6/0.5 | 3.8 (-39, 7, -4)/3.5 (48, -19, 20) |
| Precuneus | 7, 19, 31 | 1.6/0.3 | 6.0 (-13, -62, 35)/2.9 (16, -81, 41) |
| Middle/Inferior Occipital Gyrus | 17, 18, 19, 37 | 0.4/1.5 | 3.7 (-28, -95, -4)/5.9 (40, -76, -5) |
| Cuneus | 18, 19, 30 | 0.7/1.4 | 5.0 (-27, -95, 0)/4.8 (18, -99, 5) |
| Postcentral Gyrus | 1, 2, 3, 40, 43 | 1.2/0.7 | 4.6 (-65, -28, 21)/3.6 (30, -30, 66) |
| Lingual Gyrus | 17, 18, 19 | 1.1/1.5 | 4.4 (-24, -96, -3)/4.5 (18, -94, -8) |
| Posterior Cingulate | 23, 30, 31 | 1.2/0.8 | 3.4 (-24, -65, 10)/4.4 (4, -48, 23) |
| Fusiform Gyrus | 18, 19, 20, 37 | 0.4/1.7 | 4.0 (-49, -9, -25)/4.4 (52, -53, -18) |

Notes: HC, healthy controls; SCH, schizophrenia; fALFF, fractional amplitude of low-frequency fluctuations; GMV, grey matter volume; sMRI, structural magnetic resonance imaging; fMRI, functional magnetic resonance imaging.

**Results including age, gender, duration of illness and education as covariates**

*Different network characteristics*

When including age, gender, duration of illness and education as covariates, group difference in loading coefficients of IC from fMRI (DMN network) was still significant (p=0.016), but it became weak for the other IC from sMRI (cerebello-thalamo-cortical network) (p=0.092).

*The associations between identified networks and PANSS scores*

Correlational analyses were also performed to examine the associations between the identified networks and the PANSS scores by including age, gender, duration of illness and education as covariates. The significance of the results did not change. The details are as following: the cerebello-thalamo-cortical network abnormalities from sMRI were associated with PANSS negative symptoms (r = -0.445, p = 0.020), PANSS general psychopathology symptoms (r = -0.398, p = 0.040) and the PANSS total score (r = -0.522, p = 0.005). However, we did not find any significant correlation between the fMRI DMN network and the PANSS total as well as subscale scores.

When we further tested the correlations of the fMRI DMN network with each of the PANSS negative symptom items, we found that DMN network was associated with PANSS item of blunted affect (r = 0.447, p = 0.020).
